# Machine Learning Model Development for Malignant Prostate Lesion Prediction Using Texture Analysis Features from Ultrasound Shear-Wave Elastography

**DOI:** 10.3390/cancers17081358

**Published:** 2025-04-18

**Authors:** Adel Jawli, Ghulam Nabi, Zhihong Huang, Abeer J. Alhusaini, Cheng Wei, Benjie Tang

**Affiliations:** 1 Biomedical Engineering, School of Science and Engineering, Fulton Building, University of Dundee, Dundee DD1 4HN, UK; 2 Division of Imaging Sciences and Technology, School of Medicine, Ninewells Hospital, University of Dundee, Dundee DD1 9SY, UK; 3 School of Physics, Engineering and Technology, University of York, Heslington, York YO10 5DD, UK; 4 Surgical Skills Centre, Dundee Institute for Healthcare Simulation Respiratory Medicine and Gastroenterology, School of Medicine, Ninewells Hospital and Medical School, University of Dundee, Dundee DD1 9SY, UK

**Keywords:** machine learning, texture analysis, prostate cancer, ultrasound, shear-wave elastography

## Abstract

**Introduction**: Artificial intelligence (AI) is increasingly utilized for texture analysis and the development of machine learning (ML) techniques to enhance diagnostic accuracy. ML algorithms are trained to differentiate between normal and malignant conditions based on provided data. Texture feature analysis, including first-order and second-order features, is a critical step in ML development. This study aimed to evaluate quantitative texture features of normal and prostate cancer tissues identified through ultrasound B-mode and shear-wave elastography (SWE) imaging and to develop and assess ML models for predicting and classifying normal versus malignant prostate tissues. **Methodology**: First-order and second-order texture features were extracted from B-mode and SWE imaging, including four reconstructed regions of interest (ROIs) from SWE images for normal and malignant tissues. A total of 94 texture features were derived, including features for intensity, Gray-Level Co-Occurrence Matrix (GLCM), Gray-Level Dependence Length Matrix (GLDLM), Gray-Level Run Length Matrix (GLRLM), and Gray-Level Size Zone Matrix (GLSZM). Five ML models were developed and evaluated using 5-fold cross-validation to predict normal and malignant tissues. **Results**: Data from 62 patients were analyzed. All ROIs, except those derived from B-mode imaging, exhibited statistically significant differences in features between normal and malignant tissues. Among the developed models, Support Vector Machines (SVM), Random Forest (RF), and Naive Bayes (NB) demonstrated the highest performance across all ROIs. These models consistently achieved strong predictive accuracy for classifying normal versus malignant tissues. Gray Pure SWE and Gray Reconstructed images Provided the highest sensitivity and specificity in PCa prediction by 82%, 90%, and 98%, 96%, respectively. **Conclusions**: Texture analysis with machine learning on SWE-US and reconstructed images effectively differentiates malignant from benign prostate lesions, with features like contrast, entropy, and correlation playing a key role. Random Forest, SVM, and Naïve Bayes showed the highest classification performance, while grayscale reconstructions (GPSWE and GRRI) enhanced detection accuracy.

## Simple Summary

Prostate cancer remains one of the most prevalent cancers affecting men globally, making early detection critical for improved treatment outcomes. Traditional imaging techniques often face challenges in clearly distinguishing between normal and cancerous tissues. In this study, we employed artificial intelligence and machine learning to analyze prostate tissue images acquired from ultrasound and a specialized method known as shear-wave elastography (SWE). By exploring patterns and textures in these images, we trained machine learning models to accurately differentiate between healthy and malignant tissues. Our results demonstrated that machine learning models, particularly Support Vector Machines, Random Forest, and Naïve Bayes, excelled in detecting prostate cancer. This research indicates that advanced image analysis combined with artificial intelligence holds the potential to enhance the diagnosis of prostate cancer, ultimately leading to quicker and more precise assessments for both patients and healthcare providers.

## 1. Introduction

Prostate cancer (PCa) is the most frequently diagnosed cancer in men worldwide. In England, it ranks as the most common cancer among men and is the second leading cause of cancer-related deaths [1]. In 2024 [2] prostate cancer is projected to remain the most common type of cancer diagnosed in men and is also anticipated to be the second most frequent cause of cancer mortality. PCa diagnosis typically involves a combination of screening, histopathology, and medical imaging techniques.

The advancement of medical imaging techniques over the years has significantly enhanced the quality of PCa diagnoses. Ultrasound (US) and magnetic resonance imaging (MRI) are the primary imaging modalities used in PCa detection. Although MRI offers greater sensitivity than the US, it comes at a considerably higher cost and is not suitable for all patients, particularly those with pacemakers, ferromagnetic metals, or those who suffer from claustrophobia. B-mode ultrasound is the fundamental parameter for assessing lesions’ location, size, and shape. However, the identification of lesions in b-mode images depends on echogenicity, meaning that some PCa lesions may appear isoechoic, displaying a brightness similar to that of the prostate gland [3]. Another ultrasound technique frequently employed in PCa diagnosis is shear-wave elastography (SWE). This quantitative method evaluates shear-wave velocity through the application of an acoustic radiation force impulse (ARFI) to the tissue, which estimates Young’s modulus of the tissue [4]. The results are presented as a superimposed color map overlaid on each pixel of the grayscale ultrasound image [5]. SWE demonstrates higher sensitivity and specificity in detecting PCa compared to other ultrasound modalities [6]. However, it does face limitations in detection depth, which restricts its ability to measure deeper regions of the prostate [7]. Additionally, the accuracy of SWE results can be adversely affected by the presence of prostate stones or calcifications [8,9].

Recently, artificial intelligence (AI) has been increasingly utilized for texture analysis and the development of machine learning (ML) techniques to enhance diagnostic accuracy. Machine learning is a subset of AI that enables computer systems to learn patterns from data and make predictions or decisions without explicit programming. ML algorithms are trained to differentiate between normal and malignant conditions based on provided data [10]. In the realm of ultrasound imaging, the primary applications of machine learning include classification and computer-aided diagnosis, regression analysis, and tissue segmentation. Furthermore, ML is also employed in image registration and content retrieval [11]. By leveraging mathematical models, ML enhances the ability to analyze complex imaging features, improving diagnostic precision and reducing human observer variability. Texture analysis serves as a classification and segmentation tool within machine learning, providing a quantitative assessment of pixel metrics that surpass human visual capabilities [12]. The process of texture analysis involves several key steps: image acquisition, image segmentation, and feature extraction [13].

Image acquisition is a critical stage in texture analysis, as it involves selecting the appropriate imaging modality and choosing images based on specific criteria that affect the quality and relevance of the extracted texture features. Image segmentation is the process of identifying the region of interest (ROI) in medical imaging. Selecting the ROI is a vital step that influences the quantitative collection of texture data and the results of machine learning predictions. Therefore, adhering to specific criteria when selecting the ROI for segmentation ensures targeted and meaningful analysis, thereby enhancing the robustness and reliability of the texture features extracted for machine learning applications.

Texture features can be categorized as either semantic or agnostic. Semantic features are linked to morphological aspects such as shape and size, while agnostic features pertain to intensity values, including minimum, maximum, and mean. Agnostic features are further divided into first-order such as mean, variance, skewness, kurtosis, and entropy and second-order, which include the gray-level co-occurrence matrix (GLCM), gray-level Run length matrix (GLRLM), gray-level size zone matrix (GLSZM), and gray-level distance zone matrix (GLDZM) [12,13,14]. In the field of ultrasound and SWE, the application of texture analysis and machine learning has demonstrated promising results across various examinations. Morphological and first-order texture analysis features have been extracted from B-mode breast images to differentiate between different types of breast lesions effectively [15].

Quantitative ultrasound spectral analysis of B-mode breast ultrasound images utilized GLCM texture features to distinguish between malignant and normal lesions, yielding statistically significant differences across several spectral parameters [16]. Xiao et al. (2014) [17] developed a reconstruction process for SWE ultrasound images of the breast, which was assessed for the quantity of texture features. Their findings indicated a high performance in differentiating between malignant and normal conditions.

Additionally, first-order and second-order texture features were extracted from breast B-mode and SWE ultrasound images, and no statistically significant differences among all features [18]. For thyroid gland assessments, GLCM texture features derived from B-mode ultrasound images were compared with real-time elastography results [19]. In this context, purified SWE ultrasound ROIs were generated by subtracting shear-wave pixels from the B-mode thyroid gland images, facilitating enhanced extraction of GLCM texture features. The results showed a pronounced efficacy of the purified SWE images in distinguishing malignant from normal lesions [20]. Machine learning models, including logistic regression, naive Bayes, quadratic discriminant analysis, and support vector machines (SVM), have also been employed to differentiate between renal cell carcinoma and angiomyolipoma based on ultrasound shear-wave velocity [21]. GLCM texture features from ultrasound images of salivary glands were evaluated using machine learning models such as K-nearest neighbors (KNN), naive Bayes, artificial neural networks (ANN), and SVM to categorize malignant and normal conditions [22]. Prostate cancer prediction through machine learning models in ultrasound and SWE has been executed by utilizing elasticity measured in Kilopascal (kPa) as extracted features [23]. Wildeboer et al. (2020) [24] harnessed machine learning models utilizing radiomics features from ultrasound B-mode, SWE, and dynamic contrast-enhanced ultrasound to assess machine learning’s potential in this domain. In the study of Wang et al. (2022) [25], machine learning models were evaluated based on radiomics features extracted from transrectal ultrasound video clips of prostate cancer.

B-mode ultrasound and SWE are commonly used imaging modalities for PCa detection, but they have notable limitations. SWE, for instance, is influenced by factors such as prostate gland enlargement, lesion depth, and machine dependency, which can affect its diagnostic performance. Additionally, conventional imaging may not fully capture the textural characteristics differentiating malignant from normal prostate tissue. To address these challenges, this study intends to evaluate quantitative texture features of normal and prostate cancer tissues as identified through ultrasound B-mode and SWE imaging with reconstructed images. By extracting these texture features, we will develop and assess machine learning models to predict and classify normal versus malignant prostate tissue to enhance non-invasive diagnostic accuracy.

## 2. Materials and Methods

### 2.1. Patients

The East of Scotland Ethical Service approved this study, a protocol-driven study with prior ethical approval (REC ref GTCAL11197). A prospective study was approved by ethical and institutional review boards to evaluate the diagnostic accuracy of transrectal SWE ultrasound in detecting prostate cancer. Between November 2013 and August 2017, a total of 125 consecutive participants with clinically localized PCa, who opted for and were scheduled to undergo laparoscopic radical prostatectomy (LRP), were enrolled.

Transrectal ultrasound shear-wave (TRUS) SWE examinations were performed by a urologist with over 10 years of experience on the day of the LRP, using an endocavity Aixplorer^®^ ultrasound transducer inserted through the rectal wall, ensuring focus on the prostate while minimizing pressure on the transducer. Prostate sections were examined by a uropathologist with over 20 years of experience who was blinded to the SWE imaging results. Pathological findings, including the disease stage and margin status, were compared with the SWE imaging outcomes.

A total of 64 patients were excluded from the texture analysis based on specific criteria outlined in Table 1. This exclusion was due to several factors: some patients lacked results for radical prostatectomy (RP), others had no images that captured both true positive and true negative correlations with the RP result, and a number had lesions smaller than 5 mm. Patients were excluded if they did not have both true negative and true positive SWE results to ensure a balanced dataset and to avoid bias. Quantitative texture features are extracted exclusively from images with a radical prostatectomy reference size of at least 5 mm, owing to the limitations of SWE in detecting small lesions [8].

### 2.2. Image Reconstructions

**Figure 1:**
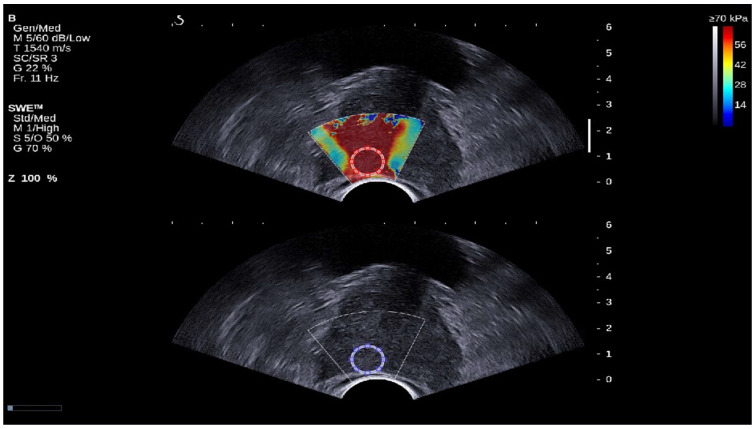
SWE images with b-mode ultrasound image of prostate cancer lesion. At the top (Red circle), the ROI is selected from the SWE image, and it is duplicated automatically in the B-mode ultrasound image (Blue circle). The red color map in the SWE image correspond to areas of higher stiffness, whereas the blue color map indicate areas of lower stiffness.

**Figure 2:**
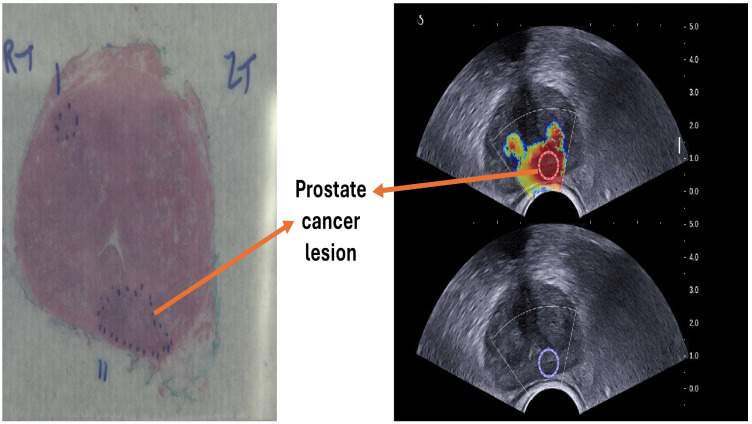
Visualization of prostate cancer lesion in the SWE and B-mode ultrasound (**Right side**), confirmed by radical prostatectomy image (**Left side**).

From 62 patients, 50 patients provide true positive and true negative images. Of these 50, 5 images also with false positive and false negative, and 12 cases have only false positive and false negative images. Two ROIs were selected, first from SWE images, and based on the result of the RP image they were already approved in the image by a urologist, and the second ROI was automatically duplicated in ultrasound b-mode image by using MATLAB code (MATLAB R2023b) to extract the image of normal tissue and PCa tissue Figure 1. This selection is based on the location of the lesion in the radical prostatectomy image Figure 2. In all images, the ROI diameter was fixed at 60 pixels. Binary masks are first created for each ROI to isolate and analyze specific ROIs within the composite SWE and b-mode image. The binary masks allow for selective extraction of each ROI by retaining only the pixel values within the region of interest while setting all other pixels to zero. To transfer these ROIs specifically to a B-mode format, the Binary singleton expansion function (bsxfun) is used to apply each binary mask across all channels of the ultrasound image [26]. This process ensures efficient extraction of ROI from both SWE and b-mode, where only the pixels inside each ROI retain their original values, while pixels outside the ROIs are zeroed. The b-mode ROI is then converted to grayscale for further analysis on a uniform grayscale image. This approach enables targeted analysis of the selected ROIs by isolating them from the surrounding image regions, ensuring focused evaluation of specific areas within the composite image.

**Figure 3:**
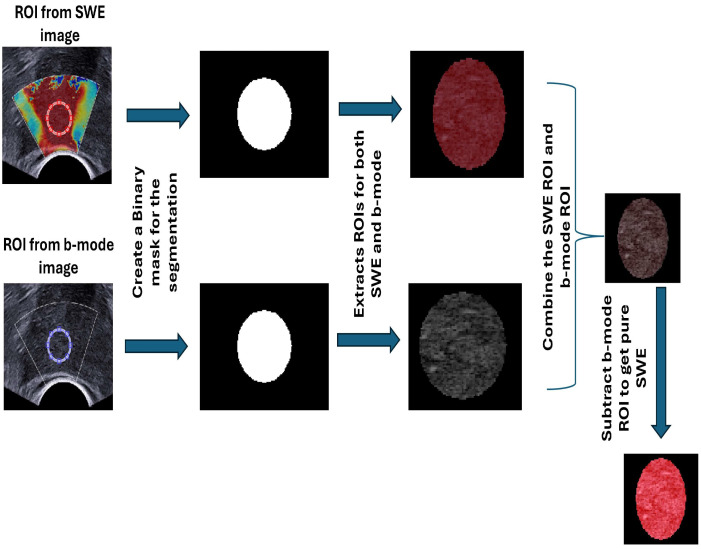
Illustration of creating a pure SWE ROI. After selecting ROIs on both SWE and b-mode ultrasound images, binary masks are created for segmentation. ROIs are extracted from both images, combined, and subtracted from the b-mode ROI to obtain the pure SWE ROI.

**Figure 4:**
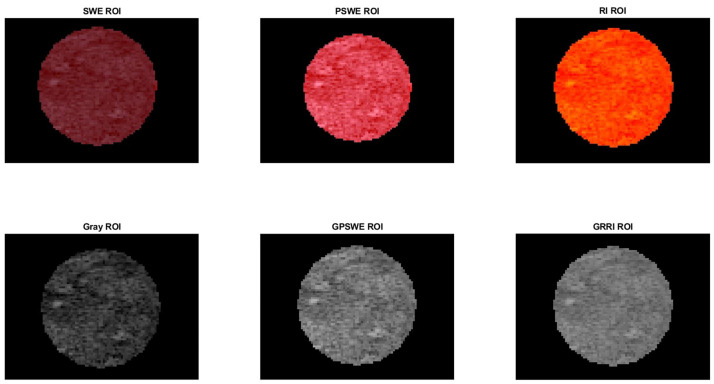
Six ROIs extracted from image of malignant lesion of prostate. The top row shows the color ROI, and the bottom rows show the Grayscale ROIs.

The SWE ROI is purified to create a pure SWE ROI by subtracting shear-wave pixels from the B-mode to obtain pure-SWE (PSWE) ROI. This is achieved by combining the SWE image and the transferred ROIs to create a composite SWE image. This composite image is generated by adding the ROI of masked version of the SWE image, SWE ROI after using (bsxfun), and the ROI of the b-mode, allowing for a comprehensive view of the selected ROIs alongside the SWE image. Subsequently, the B-mode ROI is subtracted from the composite image to isolate the SWE content, resulting in PSWE ROI [20] Figure 3. Then this is converted from 3-channel (Red, Green, Blue) RGB color to a single grayscale intensity value to obtain gray-pure-SWE ROI (GPSWE). A custom colormap is created to show specific areas in an image using a range of colors from red to blue. This starts by choosing 5500 colors for each transition to allow for smooth changes between colors, which keeps details clear in interest. Each pixel in the selected area is matched to the closest color from this colormap by using a Euclidean distance [17]. This method finds the color that best matches the pixel’s RGB values, reducing the color range in that area to a gradient of red, yellow, green, and blue to reconstruct the PSWE ROI into a new ROI, and it is called RI ROI. The RI ROI is then converted into a gray image to obtain a gray reconstructed (GRRI) ROI. Consequently, six ROIs are extracted from normal and malignant prostate areas, and their data are saved for texture analysis processing, as is shown in Figure 4.

### 2.3. Texture Analysis

Ninety-four texture analysis features from the first order and second order were selected based on the study [14] using MATLAB (R2023b) codes from all ROIs, and they are mentioned in Table 2. The features are calculated at specific angles (0°,45°,90°,135°) which represent the orientations used to evaluate certain properties or characteristics [27]. The MATLAB code is written to compute texture features; for instance, the Contrast feature is computed Contrast based on the GLCM matrix function corresponds to the following mathematical equation Equation (1) [28]

contrast = ∑ (i, j) 2q(i, j) (1)

where q(i, j) is the pixel at location (i, j).

All data are saved and labeled for begin and malignant as 0 and 1, respectively. The feature data were normalized using Z-score normalization, which involved calculating the mean and standard deviation of the features. This process cantered data by subtracting the meaning from each feature value and scaled it to have a standard deviation of one, ensuring that all features contributed equally to subsequent analyses [29]. Following normalization, statistical significance was obtained by using a *t*-test to provide the *p*-values and evaluate the differences between normal and malignant cases. The resulting *p*-values from these tests are compared against a threshold of 0.05 to determine significant features.

### 2.4. Machine Learning Models

Feature selection is one of the important steps in ML modeling. Due to the large number of features, feature selection is useful for adopting pertinent features and removing unnecessary or unrelated features. By using MATLAB codes (MATLAB R2023b) Least absolute shrinkage and selection operator (LASSO) [30] was used on the features with statistically significant differences between normal and malignant, and it helps mitigate overfitting by penalizing the absolute size of the coefficients [31]. The range of lambda values was defined, and cross-validation was utilized to identify the optimal lambda that minimizes prediction error while controlling the number of features included in the model. Consequently, the most significant features that contributed to the classification task enhancing the robustness and interpretability of our model were selected.

A systematic methodology was implemented to evaluate the performance of five different machine learning models: random forest (RF), KNN, logistic regression (LR), SVM, and naive bayes (NB), These models were selected based on their proven effectiveness in medical imaging and classification tasks [32,33,34], Two complementary validation techniques were employed to evaluate the effectiveness of the machine learning models. Initially, a hold-out validation method was applied, which involved designating 30% of the dataset as a separate test set, while the remaining 70% was used for training purposes. In addition, on a classification task on the selected features. Five k-fold cross-validation was employed, which allowed us to divide the dataset into training and testing subsets for each fold. In this approach, the entire dataset is partitioned into five distinct subsets. In each iteration, one of the subsets is utilized as the test set, while the remaining four subsets serve as the training set. This procedure is repeated for all five subsets, ensuring that each one is tested in turn [35]. The hyperparameters for each classifier were selected based on relevant research in the field of medical imaging and machine learning [36,37]. The chosen values were derived from the literature that has successfully optimized these parameters for similar classification tasks, ensuring their suitability for our dataset and problem area. For each model, the performance on the testing subset, calculating the means of the five folds with the standard deviation of metrics such as accuracy, sensitivity, specificity, and area under the receiver operating characteristic curve (AUC), was achieved. In addition, confusion matrices were provided to derive these metrics, ensuring a comprehensive evaluation of each model’s classification performance.

### 2.5. Prediction Normal and Malignant Prostate Tissue

In each ROI, except for the b-mode ultrasound, a distinct machine learning model was utilized. Specifically, the models employed for original SWE, PSWE, and GPSWE were RF and for RI ROI was SVM, and for GRRI was NB. Notably, within the PSWE and GPSWE ROI, the KNN, SVM, and NB models exhibited the highest levels of accuracy, sensitivity, and specificity. However, the RF model was ultimately selected to mitigate the risk of overfitting during the prediction process.

For the prediction, MATLAB (R2023b) was used to select and reconstruct the ROI from the ultrasound images. Relevant features were then extracted from the newly selected ROI and saved for further analysis. In the next step, to ensure compatibility with the pre-trained model, the saved feature data were loaded, referencing the feature names used during model training. Common features between the new dataset and the training data were identified and aligned, ensuring that only features present in both datasets were utilized for prediction. If no common features were identified, an error was generated.

**Figure 5:**
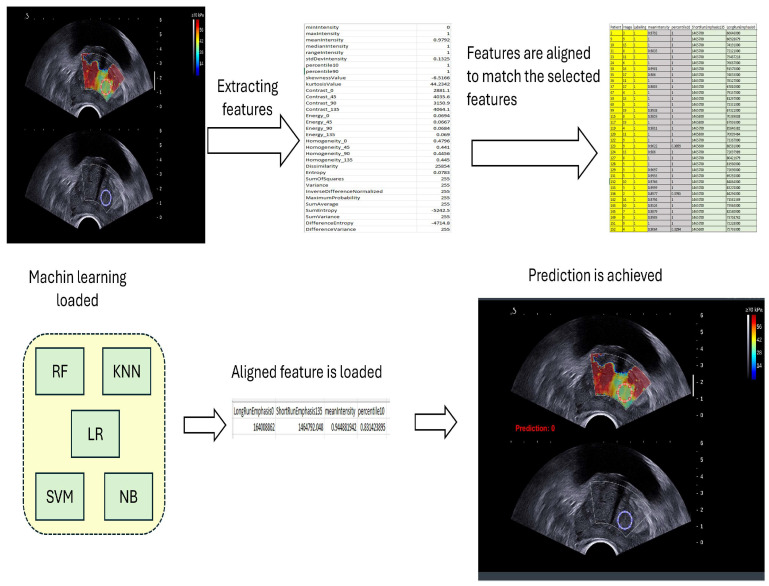
Steps of prediction processing.

Once the features from the new data set were aligned with the training data, they were appropriately formatted for input into the trained model. These aligned features were then processed through the model to generate predictions. The trained model classified the new data into predefined categories, such as “normal” or “malignant”. Ultimately, the classification results were displayed, providing predicted labels for the new samples based on the trained model, Figure 5.

## 3. Results

A study was conducted involving 62 patients diagnosed with prostate cancer, where six ROIs were extracted from both normal and malignant prostate tissue. This led to the collection of 50 images representing normal tissue and another 50 for malignant tissue. The general characteristics of the patient cohort are detailed in Table 3. In the analysis of texture features, statistical evaluation via the *t*-test revealed no significant differences between normal and malignant tissues in the Gray ROI. However, significant discrepancies were observed in the SWE ROIs, with a total of 17, 27, 41,26, and 37 features demonstrating statistically significant differences between normal and malignant cases when considering the original SWE ROI, PSWE ROI, GPSWE ROI, RI ROI, and GRRI ROI, respectively.

This investigation explores the correlation between Prostate-Specific Antigen (PSA) levels and texture features by employing one-way ANOVA. PSA levels were classified into three distinct clinical categories: normal (≤4 ng/mL), gray zone (4–8 ng/mL), and high risk (≥8 ng/mL) [38]. The objective of the statistical analysis was to assess whether the distribution of texture feature values displayed significant differences across these PSA classifications when utilizing various imaging modalities.

The results demonstrated that in Gray images, only one feature—minimum intensity—was found to be statistically significant, with a *p*-value of 0.0408. In contrast, Original SWE images did not present any significant texture features out of the 94 analyzed. Within the PSWE images, two features were statistically significant: Entropy (*p*-value = 0.0320) and Low Gray Level Run Emphasis 90 (*p*-value = 0.0448). For GPSWE images, one feature, standard deviation of intensity, exhibited statistical significance (*p*-value = 0.0204). A further analysis of RI images revealed five significant features related to PSA levels, including Energy 0 and High Gray Level Zone Emphasis across four angles (0°, 45°, 90°, and 135°), with *p*-values ranging from 0.0149 to 0.0430. Lastly, GRRI images yielded the most significant findings, particularly about standard deviation intensity and features 50–53 (High Gray Level Run Emphasis) and features 81–84 (High Gray Level Zone Emphasis), all revealing *p*-values below 0.03. These results indicate that specific texture features—especially those associated with gray-level emphasis and run length patterns—exhibit significant variability with PSA levels, suggesting that their relevance is significantly influenced by the specific image reconstruction technique used.

Simultaneously, the relationship between the Gleason Score (GS) and radiomic features was assessed using a one-way ANOVA, aiming to identify which features demonstrate significant fluctuations across various GS categories, which are stratified into grades 6 to 10. The findings revealed variability in the number and types of significant features contingent on the image reconstruction method employed. For Gray images, no statistically significant features were identified. Conversely, Original SWE images showcased nine significant features, primarily linked to contrast, entropy, and zone-based metrics. PSWE images exhibited the highest number of significant features, totaling 23, while GPSWE images revealed 21. Both RI and GRRI images identified 19 significant features each Table S1. Overall, these findings highlight a strong association between GS and a diverse range of radiomic features, particularly those associated with texture complexity and gray-level distribution, emphasizing notable disparities across different imaging modalities.

Tables S2–S4 summarize the features that exhibited significant differences between normal and malignant tissues across the original SWE ROI, pure SWE ROI, and GPSWE ROIs., respectively. Each table includes features that are classified under various classifications. Tables S5 and S6 focus on the features that showed a notably high level of significance between normal and malignant tissues in the RI ROI and GRRI ROI, specifically highlighting features that were excluded from the GLSZM classification.

**Figure 6:**
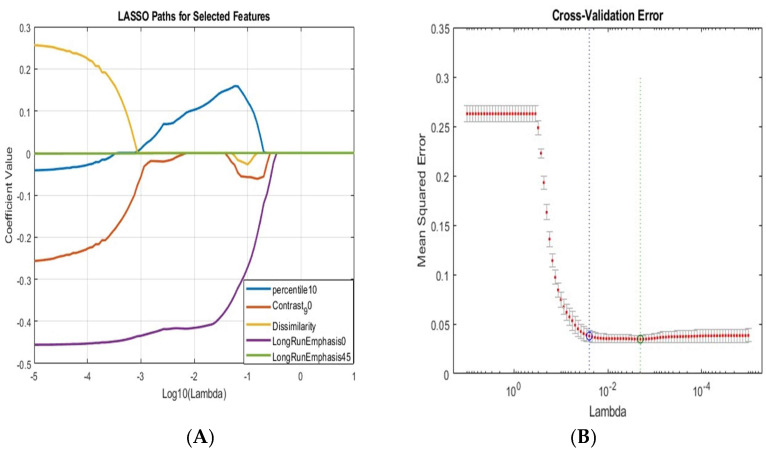
(**A**) Plot the LASSO coefficient for the selected features of the RI ROI features and the names of the features selected. (**B**) Plot of cross-validation error.

**Figure 7:**
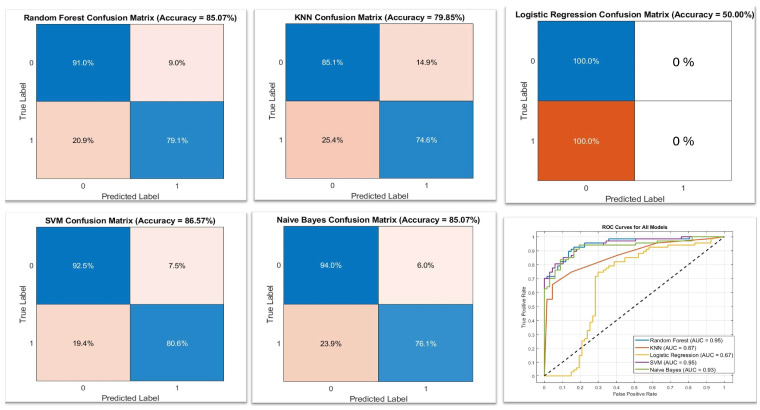
Performance Evaluation of Machine Learning Models for Classification. This figure includes confusion matrices for each model, showcasing their classification accuracy on prostate tissue within the RI ROI. The confusion matrices appear in the first row and the first two images of the second row (from the right). A comparison of model performance is visualized in the ROC curve (last image in the second row), illustrating the discriminatory ability of each model across different classification thresholds.

In the context of machine learning model development, the evaluation of model performance is crucial for ensuring reliability and accuracy. The cross-validation error and the results from LASSO regression are illustrated in Figure 6. This figure depicts the LASSO regression coefficient paths corresponding to the selected features, with feature names associated with non-zero coefficients displayed alongside each coefficient path. The features included in the model were meticulously chosen based on cross-validation techniques aimed at minimizing the mean squared error, thereby identifying the most significant predictors for the model. The specific features selected from each ROI are detailed in Table 4. Additionally, the evaluation results of the machine learning model are summarized in Figure 7.

Table 5 includes key metrics such as Sensitivity, Specificity, and Accuracy. In comparing the various models, confusion matrices serve to illustrate the accuracy of each model in predicting both positive and negative cases of prostate tissue. Furthermore, the receiver operating characteristic (ROC) curves provide a visual representation of model performance, where higher AUC values are indicative of superior discriminatory capabilities, as shown in Figure 7.

Original SWE: the original image of the SWE, PSWE: is the purified image of the original SWE, GPSWE: is the gray image of the PSWE. RI: is the reconstructed image of the original SWE. GRRI: is the gray image of the RI.

**Figure 8:**
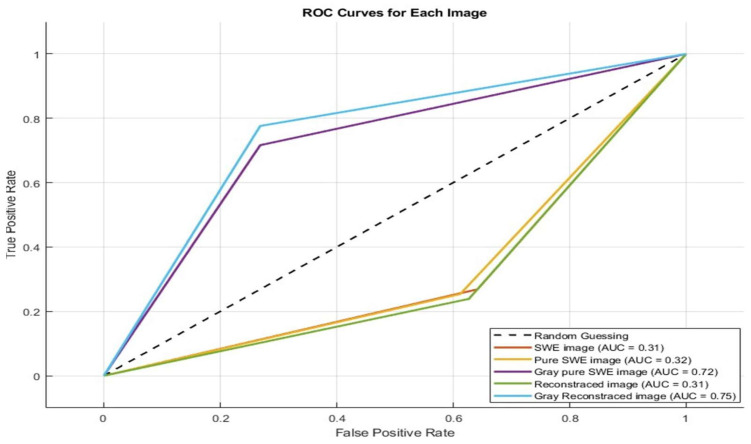
ROC curve of the machine learning models to predict the normal and malignant in true positive and true negative images.

The predictions were applied to the same data used to extract quantitative features for true positives and true negatives. The performance metrics of the machine learning model in predicting normal and malignant prostate cancer cases for all images are presented in Table 6, along with the ROC curve shown in Figure 8. Table 7 and Table 8 show the performance metrics of the machine learning model in predicting normal and malignant prostate cancer cases for images with true positive and negative and false positive and negative, respectively.

## 4. Discussion

Our primary objective was to create machine learning models utilizing several reconstructed images of SWE from both prostate cancer and normal tissues. These models were designed to accurately predict the classification of normal and malignant tissues within SWE prostate imaging.

In this study, we assessed 94 features extracted from both normal and malignant prostate lesions using B-mode ultrasound and SWE. We successfully reconstructed ROIs from SWE images. The reconstruction of PSWE and RI ROIs was accomplished effectively, as evidenced by the distinct quantitative features obtained from each ROI. The differences in feature values among the original SWE, PSWE, and RI images further confirm the integrity and effectiveness of the reconstruction process. Moreover, the grayscale representations of the GPSWE and GRRI images exhibited clear variations compared to the original B-mode image, validating the successful transformation and extraction of unique quantitative and textural information. These findings underscore the potential of reconstructed ROIs to deliver complementary diagnostic insights that extend beyond traditional B-mode and SWE imaging techniques.

Despite the advanced capabilities of modern B-mode imaging, none of the features extracted from this method showed statistical significance in differentiating normal from malignant prostate lesions in our analysis. This outcome is consistent with [39], which underscores the inherent limitations of B-mode ultrasound in distinguishing between normal and malignant prostate tissues. B-mode imaging primarily offers anatomical and structural insights, failing to capture the subtle tissue characteristic differences associated with malignancy. Prostate cancer is widely recognized for its heterogeneity, and the overlapping echotexture and grayscale intensity between normal and malignant lesions render differentiation particularly challenging [40]. Additionally, factors such as glandular distortion, calcifications, and normal prostatic hyperplasia (BPH) further complicate the interpretation of b-mode ultrasound.

The differentiation between normal and malignant lesions is informed by several significant features identified in ultrasound shear-wave imaging. This imaging technique captures variations in tissue stiffness and spatial patterns, proving valuable for the differential diagnosis of lesions. Notably, GLCM features such as “Contrast” and “Homogeneity” are instrumental in illustrating the heterogeneity and uniformity of tissue stiffness. Higher contrast values often indicate malignant regions, thereby enhancing the diagnostic potential of this imaging modality [18]. Features, including “High Gray Level Zone Emphasis” and “Long Run Emphasis”, play a crucial role in identifying extensive zones of high stiffness, which are reflective of pathological changes associated with malignancy. Intensity-based metrics, such as “Mean Intensity” and “Minimum Intensity”, further contribute to the assessment by quantifying overall stiffness; malignancies generally present with elevated mean stiffness levels compared to normal tissues. Together, these features leverage the color-coded shear-wave elasticity data to characterize the mechanical properties of prostate tissue, providing robust differentiation between normal and malignant lesions.

In contrast, features extracted from grayscale images provide a significantly larger dataset for analysis, presenting both advantages and challenges in distinguishing between normal and malignant lesions. Grayscale images typically capture more detailed variations in texture, highlighting finer nuances in tissue heterogeneity and intensity distribution. This results in a wider array of features, such as those obtained from GLCM, GLRLM, GLSZM, and pixel intensity metrics, which enhance the ability to differentiate subtle variations between tissue types [41]. These additional features can bolster the model’s discriminatory power by offering a more comprehensive characterization of tissue stiffness and structure. However, the increased feature set introduces challenges, particularly in the development of machine learning models. With a larger number of features, there is a heightened risk of overfitting, especially when the training data are limited [42].

It has been observed that the GLSZM features, which are sensitive to heterogeneity, show a lack of significance with the RI and GRRI. This indicates that the texture information essential for distinguishing between normal and malignant tissues may have been lost or diminished during the reconstruction process. Specifically, GLSZM features, which depend on identifying variations in the size and distribution of homogeneous regions, may not effectively capture subtle heterogeneities when the image has been excessively smoothed or homogenized. This absence of significant differentiation suggests that the reconstructed ROI may have become overly uniform or noisy, thereby obscuring the intricate textural patterns often characteristic of malignant tissues [43]. These patterns, including irregular zone sizes and varying intensities, are vital for differentiating malignant lesions from normal ones. Consequently, the smoothing effects during reconstruction may have impaired the GLSZM’s ability to identify key pathological features, potentially explaining the non-significant findings in the analysis.

The machine learning models generally demonstrate strong performance across the five ROIs, with SVM, KNN, and NB achieving perfect results in the original SWE and PSWE ROI. This indicates that the features within this region are linearly separable, enabling the models to data file. However, this success may also raise concerns regarding overfitting, which should be assessed using an independent test set. This observation can be compared with studies [23,25], which typically indicate a more cautious approach when analyzing larger and more diverse patient populations. It is crucial to acknowledge that the number of patient data points is vital for achieving reliable and generalizable model effectiveness. Insufficient sample sizes can result in overly optimistic outcomes that may not be applicable in real-world clinical settings. For example, in our study, the logistic regression sensitivity and specificity for the original SWE were 0% and 100%, respectively, where it was 61.1% and 91.1%, respectively [23]. Conversely, LR struggles in the original SWE, PSWE, and GPSWE ROI, likely due to its linear characteristics that fail to capture the non-linear relationships present in this area.

The results of the machine learning models reveal interesting performance trends across different image preprocessing techniques and categories of cases (true positive, true negative, false positive, and false negative). Gray Pure SWE and Gray Reconstructed images consistently outperformed other methods, achieving sensitivities, specificities, and accuracies of 71.6–98%, 73.1–96%, and 72.4–97%, respectively. These findings suggest that converting images to grayscale enhances texture analysis by capturing more discriminative features, leading to improved classification of normal and malignant tissues. Conversely, SWE images and reconstructed images in their raw forms demonstrated poor performance, with sensitivities, specificities, and accuracies below 40% in most cases. This discrepancy highlights the importance of preprocessing techniques in enhancing model performance.

When comparing ML and deep learning (DL), the key difference lies in how each approach handles feature extraction and learning from data. ML algorithms, such as SVM, KNN, and RF, depend on manually selected features. This reliance on feature engineering makes ML methods more interpretable and effective for smaller datasets.

In contrast, deep learning, particularly through convolutional neural networks (CNNs), automatically extracts hierarchical features from raw data. This capability often results in superior performance on complex image analysis tasks. However, deep learning requires large datasets and significant computational resources, making it more susceptible to overfitting when working with limited data [44].

While machine learning remains a viable option in scenarios with constrained datasets, future research may explore hybrid approaches that combine ML feature extraction with DL architectures to enhance both performance and reliability.

In a recent application, deep learning was used to distinguish between prostate cancer and benign prostatic hyperplasia, utilizing a vast number of transrectal ultrasound (TRUS) images. The performance of CNNs in differentiating between benign and malignant prostate cancer was notably high [45].

When analyzing subsets of data, such as only true positive/true negative cases or false positive/false negative cases, we observed further disparities. SWE and Pure SWE excelled in identifying false positive and false negative cases, achieving sensitivity and specificities as high as 100%. However, these models failed in the general classification tasks, with an accuracy of below 10% for true positive and true negative cases. Gray images, in contrast, performed exceptionally well for true positive/true negative cases but struggled with false positive/false negative cases, where sensitivities and specificities dropped to as low as 18%.

The observed trends might stem from the small dataset size, particularly the limited number of false positive and false negative cases. A small sample size can lead to biased learning and limited generalization capacity for the models, particularly in imbalanced or borderline scenarios. Future studies with larger datasets are needed to validate these findings and improve robustness. The machine learning classifiers developed in this study show promising results; however, several limitations may affect their overall performance. Firstly, the limited number of cases could impede the models’ ability to generalize effectively. Smaller datasets often result in overfitting, biased outcomes, and reduced stability, highlighting the need for larger and more diverse datasets to strengthen the robustness and reliability of the classifiers. Additionally, the data were obtained from the Axiplorer machine (Supersonic Imagine in Aix-en-Provence, France), which has limitations concerning heterogeneous lesions [46]. Furthermore, the dataset is outdated, and newer machines offer superior image quality. Another limitation of this study is that the texture analysis and machine learning models were built from a high number of true positive and true negative images compared with false positive and false negative images. This selective method introduces bias, as the models are trained exclusively on instances with clear classifications. Consequently, they may encounter difficulties in accurately classifying cases that involve false positives or false negatives. This challenge is further intensified by the inherent limitations of SWE ultrasound, where image quality and feature representation may not always be adequate to address ambiguous or borderline cases. As a result, this may hinder the models’ ability to generalize to more complex real-world scenarios. Additionally, the performance of the developed classifiers was not compared with existing state-of-the-art methods, which limits the contextual evaluation of their effectiveness. Furthermore, the models were only tested on a single dataset and were not externally validated on independent datasets. This limits the generalizability of the findings and highlights the need for future studies to evaluate the models on diverse data sources.

## 5. Conclusions

Texture analysis utilizing machine learning on SWE and reconstructed images serves as an objective and valuable method for distinguishing between normal and malignant prostate lesions. Among the extracted texture features, contrast, entropy, correlation, and homogeneity were particularly effective in differentiating malignant from benign lesions. RF, SVM, and NB demonstrated the highest classification performance, with [mention accuracy, AUC, or other performance metrics if available]. Notably, reconstructing SWE into grayscale images, such as GPSWE and GRRI, significantly enhanced prostate cancer detection accuracy using machine learning.

## Figures and Tables

**Table 1:** Exclusion criteria.

Criteria	Number of Excluded Patients
Small lesion < 5 mm	19
No laparoscopic radical prostatectomy results	13
No true positive SWE result	12
No true Negative SWE result	12
No SWE detection	7

**Table 2:** First and second-order features used for assessing the quantitative texture analysis of the prostate ultrasound and SWE images.

Classification	Categories	Features
**First-order**	Intensity	Minimum Intensity
		Maximum Intensity
		Mean Intensity
		Median Intensity
		Range Intensity
		Standard deviation Intensity
		Percentile 10
		Percentile 90
		Skewness Value
		Kurtosis Value
**Second-order**	GLCM	Contrast (0°,45°,90°,135°)
		Energy (0°,45°,90°,135°)
		Homogeneity (0°,45°,90°,135°)
		Dissimilarity
		Entropy
		Sum of Squares
		Variance
		Inverse Difference Normalized
		Maximum Probability
		Sum Average
		Sum Entropy
		Sum Variance
		Difference Entropy
		Difference Variance
	GLRLM	Short Run Emphasis (0°,45°,90°,135°)
		Long Run Emphasis (0°,45°,90°,135°)
		Gray Level Non-uniformity (0°,45°,90°,135°)
		Short Run Low Gray Level Emphasis (0°,45°,90°,135°)
		High Gray Level Run Emphasis (0°,45°,90°,135°)
		Low Gray Level Run Emphasis (0°,45°,90°,135°)
		Run Percentage (0°,45°,90°,135°)
		Run Length Non-Uniformity
	GLDZM	Zone Percentage (0°,45°,90°,135°)
		Gray Level Non-uniformity (0°,45°,90°,135°)
		Zone Size Non-uniformity (0°,45°,90°,135°)
		Zone Size Variance
		Zone Entropy
		Low Gray Level Zone Emphasis (0°,45°,90°,135°)
		High Gray Level Zone Emphasis (0°,45°,90°,135°)
		Gray Level Non-Uniformity Normalized (0°,45°,90°,135°)
		Zone Size Non-Uniformity Normalized
	GLSZM	Size Zone Non-uniformity
		Zone Percentage
		Gray Level Non-uniformity
		Run Length Non-uniformity
		Run Percentage

GLCM: Gray-Level Co-Occurrence Matrix, GLDLM: Gray-Level Dependence Length Matrix, GLRLM: Gray-Level Run Length Matrix, GLSZM: Gray-Level Size Zone Matrix.

**Table 3:** Selected patient characteristics.

No. Pts	62
Mean ± SD age/median	68 ± 5.5/67
Mean ± SD PSA/median	12 ± 8/9
No. Gleason Score	
3 + 3	0
3 + 4	30
4 + 3	12
3 + 5	7
4 + 4	1
4 + 5	12

**Table 4:** Feature selected by LASSO for each ROI.

ROIs	Features Selected
**Original SWE**	Homogeneity 90°
	Long Run Emphasis 45°
	Long Run Emphasis 135°
**PSWE**	Long Run Emphasis 0°
	Long Run Emphasis 45°
	Long Run Emphasis 90°
**GPSWE**	Homogeneity 0°
	Variance
**RI**	Percentile 10
	Contrast 90°
	Dissimilarity
	Long Run Emphasis 0°
**GRRI**	Median Intensity
	Contrast 0°
	Inverse Difference Normalized

**Table 5:** Evaluation Metrics for the Machine Learning Model for the ROIs.

ROI	Model	Sensitivity % ± SD	Specificity % ± SD	Accuracy % ± SD
**Original SWE**	Random Forest	98.75 ± 2.80	97.65 ± 5.26	97.78 ± 3.31
	KNN	100.00 ± 0.00	97.65 ± 5.26	98.52 ± 3.31
	Logistic Regression	0.00 ± 0.00	100.00 ± 0.00	50.00 ± 8.49
	SVM	100.00 ± 0.00	97.65 ± 5.26	98.52 ± 3.31
	Naive Bayes	100.00 ± 0.00	97.65 ± 5.26	98.52 ± 3.31
**PSWE**	Random Forest	98.75 ± 2.80	97.65 ± 5.26	97.78 ± 3.31
	KNN	100.00 ± 0.00	97.65 ± 5.26	98.52 ± 3.31
	Logistic Regression	0.00 ± 0.00	100.00 ± 0.00	50.00 ± 8.49
	SVM	100.00 ± 0.00	97.65 ± 5.26	98.52 ± 3.31
	Naive Bayes	100.00 ± 0.00	97.65 ± 5.26	98.52 ± 3.31
**GPSWE**	Random Forest	85.53 ± 13.23	71.59 ± 11.07	77.58 ± 6.03
	KNN	85.53 ± 13.23	68.03 ± 6.84	76.10 ± 6.82
	Logistic Regression	0.00 ± 0.00	100.00 ± 0.00	50.06 ± 9.68
	SVM	82.33 ± 14.74	73.02 ± 10.33	76.84 ± 6.75
	Naive Bayes	83.99 ± 11.46	71.68 ± 10.70	76.84 ± 6.75
**RI**	Random Forest	73.80 ± 13.95	91.16 ± 6.01	82.14 ± 8.76
	KNN	76.82 ± 12.50	68.69 ± 15.74	72.42 ± 8.40
	Logistic Regression	52.31 ± 50.27	55.38 ± 51.43	50.88 ± 10.42
	SVM	83.08 ± 16.69	92.49 ± 5.48	87.41 ± 10.99
	Naive Bayes	76.56 ± 10.13	95.59 ± 6.78	85.87 ± 6.55
**GRRI**	Random Forest	75.30 ± 8.83	69.89 ± 13.97	73.16 ± 10.19
	KNN	69.92 ± 7.77	71.95 ± 9.90	70.88 ± 6.68
	Logistic Regression	0.00 ± 0.00	100.00 ± 0.00	50.06 ± 10.36
	SVM	80.88 ± 7.29	67.30 ± 10.91	74.64 ± 7.05
	Naive Bayes	82.20 ± 8.03	67.01 ± 13.40	75.38 ± 9.25

**Table 6:** Performance metrics of the prostate cancer detection model for all selected images.

Image	TP	TN	FP	FN	Sensitivity	Specificity	Accuracy
**SWE Image**	18	24	43	49	26.9%	35.8%	31.3%
**Pure SWE image**	17	26	41	50	25.4%	38.8%	32.1%
**Gray Pure SWE**	48	49	18	19	71.6%	73.1%	72.4%
**Reconstructed image**	16	25	42	51	23.9%	37.3%	30.6%
**Gray Reconstructed image**	52	49	18	15	77.6%	73.1%	75.4%

**Table 7:** Performance metrics of the prostate cancer prediction model for only images with true positive and true negative.

Image	TP	TN	FP	FN	Sensitivity	Specificity	Accuracy
**SWE Image**	1	9	41	49	2%	18%	10%
**Pure SWE image**	0	9	41	50	0%	18%	9%
**Gray Pure SWE**	41	45	5	9	82%	90%	86%
**Reconstructed image**	0	8	42	50	0%	16%	8%
**Gray Reconstructed image**	49	48	2	1	98%	96%	97%

**Table 8:** Performance metrics of the prostate cancer prediction model for only images with false positives and false negatives.

Image	TP	TN	FP	FN	Sensitivity	Specificity	Accuracy
**SWE Image**	17	15	2	0	100%	88%	94%
**Pure SWE image**	17	17	0	0	100%	100%	100%
**Gray Pure SWE**	7	4	13	10	41%	24%	32%
**Reconstructed image**	16	17	0	1	94%	100%	97%
**Gray Reconstructed image**	3	1	16	14	18%	6%	12%

## Data Availability

The data provided for the patients are available from the study are available on request from the corresponding authors and it is under the Caldicott approval number (IGTCAL11197).

## Supplementary Materials

The following supporting information can be downloaded at: https://www.mdpi.com/article/10.3390/cancers17081358/s1, Table S1: Significant Texture Features Associated with Gleason Grades via One-Way ANOVA; Table S2: Texture features with significant statistical differences between malignant and normal cases in the Original SWE ROI; Table S3: Texture features with significant statistical differences between malignant and normal cases in the Pure SWE ROI; Table S4: Texture features with significant statistical differences between malignant and normal cases in the Gray Pure SWE ROI; Table S5: Texture features with significant statistical differences between malignant and normal cases in the Reconstructed SWE ROI; Table S6: Texture features with significant statistical differences between malignant and normal cases in the Gray reconstructed SWE ROI.
