# Supplementary material for: Machine Learning Model Development for Malignant Prostate Lesion Prediction Using Texture Analysis Features from Ultrasound Shear-Wave Elastography
Source: Cancers (Basel). 2025 Apr 18;17(8):1358. doi: 10.3390/cancers17081358 (PMC12026400; doi:10.3390/cancers17081358)
Supplement: Supplementary file 1 [file cancers-17-01358-s001.zip › cancers-3517386-supplementary.pdf]

Supplementary Materials: Machine Learning Model Development for Malignant Prostate Lesion Prediction Using Texture Analysis Features from Ultrasound Shear-Wave Elastography

Adel Jawli, Ghulam Nabi, Zhihong Huang, Abeer Alhusaini, Cheng Wei and Benjie Tang

Table S1. Significant Texture Features Associated with Gleason Grades via One-Way ANOVA.

Image Type	Feature Name	p-value
**Original SWE**	Contrast 45°	0.0434
	Sum Entropy	0.0005
	Difference Entropy	0.0009
	Zone Percentage (0°,45°,90°,135°)	0.0026–0.0048
	Zone Entropy	0.0010
	Run Percentage	0.0071
**PSWE**	Contrast (0°,45°,90°,135°)	0.0086–0.0378
	Energy (0°,45°,90°,135°)	0.0006–0.0252
	Homogeneity (45°,90°,135°)	0.0028–0.0042
	Entropy	0.0079
	Sum Variance	0.0390
	High Gray Level Run Emphasis (45°,90°,135°)	0.0014–0.0022
	Zone Entropy	0.0037
	High Gray Level Zone Emphasis (45°,90°,135°)	0.0038–0.0043
**GPSWE**	Standard deviation Intensity	0.0123
	Percentile 90	0.0223
	Contrast (0°,45°,90°,135°)	0.0055–0.0189
	Entropy	0.0018
	Sum Of Squares	0.0071
	Variance	0.0247
	Sum Entropy	0.0010
	Sum Variance	0.0247
	Difference Entropy	0.0047
	High Gray Level Run Emphasis (0°,45° ,90°)	0.0238–0.0498
	Zone Percentage (45°,90°,135°)	0.0020–0.0028
	Zone Entropy	0.0012
	Run Percentage	0.0020
**RI**	Contrast (0°,45°)	0.0282–0.0297
	Energy (0°,45°,90°,135°)	0.0092–0.0231
	Homogeneity (45°–135°)	0.0213–0.0449
	Sum Entropy	0.0035
	Difference Entropy	0.0054
	Zone Percentage (0°,45°,90°,135°)	0.0282–0.0492
	High Gray Level Zone Emphasis (0°,45°,90°,135°)	0.0103–0.0201
**GRRI**	Mean Intensity	0.0304
	Standard deviation Intensity	0.0058

	Percentile 90	0.0105
	Contrast (0°,45°,90°,135°)	0.0082–0.0186
	Entropy	0.0107
	Sum Of Squares, Variance	0.0098–0.0164
	Sum Average	0.0278
	Sum Variance	0.0290
	Zone Percentage (0°,45°,90°,135°)	0.0127–0.0229
	High Gray Level Zone Emphasis (0°–90°)	0.0437–0.0493

Table S2. Texture features with significant statistical differences between malignant and normal cases in the Original SWE ROI, including mean, standard deviation, and t-test results.

Classification	Features	Mean Normal ± SD	Mean Malignant ± SD	p-Value
**Intensity**	min Intensity	35.60 ± 26.10	26.10 ± 18.50	0.2
	mean Intensity	114.30 ± 24.90	88.00 ± 20.60	0
	median Intensity	81.40 ± 7.90	75.90 ± 9.20	0
	percentile10	73.90 ± 15.30	61.80 ± 12.10	0
	skewness Value	-0.10 ± 1.10	0.80 ± 1.00	0
**GLCM**	Contrast 0°	1163.20 ± 291.30	910.90 ± 307.30	0
	Contrast 45°	1836.70 ± 415.60	1462.20 ± 455.60	0
	Contrast 90°	1236.00 ± 283.70	1005.10 ± 327.40	0
	Contrast 135°	1824.30 ± 437.20	1455.80 ± 454.50	0
	Homogeneity 0°	0.30 ± 0.00	0.30 ± 0.00	0
	Homogeneity 45°	0.30 ± 0.00	0.30 ± 0.00	0
	Homogeneity 90°	0.30 ± 0.00	0.30 ± 0.00	0
	Homogeneity 135°	0.30 ± 0.00	0.30 ± 0.00	0
**GLRLM**	Long Run Emphasis 0°	$8.70 \times 10^7 \pm 1.50 \times 10^7$	$4.50 \times 10^7 \pm 1.60 \times 10^7$	0
	Long Run Emphasis 45°	$8.70 \times 10^7 \pm 1.50 \times 10^7$	$4.50 \times 10^7 \pm 1.60 \times 10^7$	0
	Long Run Emphasis 90°	$8.70 \times 10^7 \pm 1.50 \times 10^7$	$4.50 \times 10^7 \pm 1.60 \times 10^7$	0
	Long Run Emphasis 135°	$8.70 \times 10^7 \pm 1.50 \times 10^7$	$4.50 \times 10^7 \pm 1.60 \times 10^7$	0

Table S3. Texture features with significant statistical differences between malignant and normal cases in the Pure SWE ROI, including mean, standard deviation, and t-test results.

Classification	Features	Mean Normal ± SD	Mean Malignant ± SD	p-Value
**intensity**	Mean Intensity	193.05 ± 33.02	159.22 ± 24.81	0
**GLCM**	Contrast 0°	3138.36 ± 1096.86	1937.72 ± 896.54	0
	Contrast 45°	4621.96 ± 1528.56	2951.79 ± 1285.94	0
	Contrast 90°	3284.25 ± 1073.32	2076.61 ± 910.52	0
	Contrast 135°	4638.10 ± 1565.06	2908.62 ± 1225.73	0
	Energy 0°	0.05 ± 0.02	0.02 ± 0.02	0
	Energy 45°	0.05 ± 0.02	0.03 ± 0.02	0
	Energy 90°	0.05 ± 0.02	0.03 ± 0.02	0
	Energy 135°	0.05 ± 0.02	0.02 ± 0.02	0
	Homogeneity 0°	0.43 ± 0.06	0.36 ± 0.06	0
	Homogeneity 45°	0.39 ± 0.07	0.32 ± 0.06	0
	Homogeneity 90°	0.40 ± 0.07	0.33 ± 0.06	0
	Homogeneity 135°	0.39 ± 0.07	0.32 ± 0.06	0
	Dissimilarity	34250.25 ± 4869.65	38314.91 ± 5219.36	0
	Entropy	0.12 ± 0.04	0.16 ± 0.03	0
	Sum Entropy	-5266.45 ± 745.11	-5812.95 ± 797.26	0
	Sum Variance	241.31 ± 47.64	218.51 ± 70.32	0.03
	Difference Entropy	-4677.92 ± 433.73	-4890.56 ± 559.75	0.02

GLRLM	Long Run Emphasis 0°	$1.41 \times 10^8 \pm 1.66 \times 10^7$	$7.26 \times 10^7 \pm 2.14 \times 10^7$	0
	Long Run Emphasis 45°	$1.41 \times 10^8 \pm 1.66 \times 10^7$	$7.26 \times 10^7 \pm 2.14 \times 10^7$	0
	Long Run Emphasis 90°	$1.41 \times 10^8 \pm 1.66 \times 10^7$	$7.26 \times 10^7 \pm 2.14 \times 10^7$	0
	Long Run Emphasis 135°	$1.41 \times 10^8 \pm 1.66 \times 10^7$	$7.26 \times 10^7 \pm 2.14 \times 10^7$	0
GLDM	High Gray Level Zone Emphasis 0°	0.01 ± 0.01	0.02 ± 0.03	0
	High Gray Level Zone Emphasis 45°	0.00 ± 0.01	0.02 ± 0.03	0
	High Gray Level Zone Emphasis 90°	0.01 ± 0.01	0.02 ± 0.03	0
	High Gray Level Zone Emphasis 135°	0.00 ± 0.01	0.02 ± 0.03	0
GLSZM	Run Percentage	0.01 ± 0.00	0.01 ± 0.00	0

Table S4. Texture features with significant statistical differences between malignant and normal cases in the Gray Pure SWE ROI, including mean, standard deviation, and t-test results.

Classification	Features	Mean Normal ± SD	Mean Malignant ± SD	p-Value
Intensity	Minimum Intensity	95.03 ± 52.38	69.19 ± 27.39	0
	Maximum Intensity	250.00 ± 13.02	243.09 ± 20.80	0.02
	Mean Intensity	194.90 ± 38.32	154.64 ± 29.30	0
	Median Intensity	195.67 ± 41.70	151.01 ± 32.16	0
	Range Intensity	154.97 ± 52.65	173.90 ± 34.21	0.01
	Standard deviation Intensity	25.08 ± 11.99	31.31 ± 11.44	0
	percentile10	165.45 ± 47.55	118.85 ± 31.19	0
	percentile90	224.14 ± 32.39	194.41 ± 34.48	0
	Skewness Value	-0.69 ± 1.50	0.41 ± 1.16	0
	Kurtosis Value	7.17 ± 7.19	4.89 ± 3.66	0.02
GLCM	Contrast 0°	3.63 ± 0.88	2.71 ± 0.81	0
	Contrast 45°	5.22 ± 1.11	4.05 ± 1.12	0
	Contrast 90°	3.74 ± 0.77	2.90 ± 0.76	0
	Contrast 135°	5.24 ± 1.20	4.00 ± 1.13	0
	Homogeneity 0°	1.00 ± 0.00	1.00 ± 0.00	0
	Homogeneity 45°	1.00 ± 0.00	1.00 ± 0.00	0
	Homogeneity 90°	1.00 ± 0.00	1.00 ± 0.00	0
	Homogeneity 135°	1.00 ± 0.00	1.00 ± 0.00	0
	Dissimilarity	39985.19 ± 5933.57	44031.91 ± 6457.86	0
	Entropy	0.14 ± 0.03	0.16 ± 0.03	0
	Sum Of Squares	$5.33 \times 10^6 \pm 1.30 \times 10^6$	$3.99 \times 10^6 \pm 1.19 \times 10^6$	0
	Variance	$7.61 \times 10^8 \pm 3.24 \times 10^7$	$7.26 \times 10^8 \pm 2.34 \times 10^7$	0
	Inverse Difference Normalized	$1.47 \times 10^6 \pm 220.00$	$1.47 \times 10^6 \pm 153.38$	0
	Sum Average	$4.01 \times 10^6 \pm 2.09 \times 10^5$	$3.79 \times 10^6 \pm 1.63 \times 10^5$	0
	Sum Entropy	$-3.00 \times 10^7 \pm 2147.05$	$-3.00 \times 10^7 \pm 1429.49$	0
	Sum Variance	$2.37 \times 10^{19} \pm 2.41 \times 10^{18}$	$2.11 \times 10^{19} \pm 1.83 \times 10^{18}$	0
	Difference Entropy	$-3.00 \times 10^7 \pm 1574.30$	$-3.00 \times 10^7 \pm 938.95$	0
	Difference Variance	$2.63 \times 10^6 \pm 756000.00$	$1.99 \times 10^6 \pm 669000.00$	0
GLRLM	High Gray Level Run Emphasis 0°	0.00 ± 0.00	0.00 ± 0.00	0
	High Gray Level Run Emphasis 45°	0.00 ± 0.00	0.00 ± 0.00	0.01
	High Gray Level Run Emphasis 90°	0.00 ± 0.00	0.00 ± 0.00	0
	High Gray Level Run Emphasis 135°	0.00 ± 0.00	0.00 ± 0.00	0
GLDM	Zone Percentage 0°	0.00 ± 0.00	0.01 ± 0.00	0
	Zone Percentage 45°	0.01 ± 0.00	0.01 ± 0.00	0
	Zone Percentage 90°	0.01 ± 0.00	0.01 ± 0.00	0
	ZonePercentage135°	0.01 ± 0.00	0.01 ± 0.00	0
	Zone Entropy	$-8.32 \times 10^7 \pm 5503.63$	$-8.32 \times 10^7 \pm 3472.65$	0
	High Gray Level Zone Emphasis 0°	0.00 ± 0.00	0.00 ± 0.00	0

	High Gray Level Zone Emphasis 45°	0.00 ± 0.00	0.00 ± 0.00	0
	High Gray Level Zone Emphasis 90°	0.00 ± 0.00	0.00 ± 0.00	0
**GLSZM**	Run Percentage	0.02 ± 0.01	0.02 ± 0.01	0

Table S5. Texture features with significant statistical differences between malignant and normal cases in the RI ROI, including mean, standard deviation, and t-test results.

Classification	Features	Mean Normal ± SD	Mean Malignant ± SD	p-Value
**Intensity**	Minimum Intensity	0.12 ± 0.30	0.27 ± 0.44	0.02
	Mean Intensity	0.58 ± 0.29	0.81 ± 0.30	0
	Median Intensity	0.58 ± 0.34	0.81 ± 0.33	0
	Range Intensity	0.86 ± 0.31	0.71 ± 0.44	0.02
	Standard deviation Intensity	0.23 ± 0.14	0.12 ± 0.11	0
	percentile10	0.30 ± 0.44	0.72 ± 0.43	0
**GLCM**	Contrast 0°	4451.93 ± 1340.89	3196.48 ± 899.12	0
	Contrast 45°	6294.22 ± 1754.09	4593.60 ± 1221.00	0
	Contrast 90°	4988.10 ± 1587.28	3430.31 ± 1100.79	0
	Contrast 135°	6302.58 ± 1846.80	4581.81 ± 1260.42	0
	Homogeneity 0°	0.54 ± 0.07	0.50 ± 0.05	0
	Homogeneity 45°	0.48 ± 0.06	0.46 ± 0.04	0.05
	Homogeneity 90°	0.50 ± 0.07	0.48 ± 0.04	0.01
	Homogeneity 135°	0.48 ± 0.07	0.46 ± 0.04	0.05
	Dissimilarity	45932.88 ± 17777.01	31179.10 ± 12967.70	0
	Sum Entropy	-6854.50 ± 1890.66	-5515.39 ± 1311.22	0
	Difference Entropy	-6205.66 ± 1633.05	-5050.35 ± 1143.38	0
**GLRLM**	Short Run Emphasis 0°	$1.47 \times 10^6 \pm 321.22$	$1.47 \times 10^6 \pm 280.74$	0
	Short Run Emphasis 45°	$1.47 \times 10^6 \pm 320.93$	$1.47 \times 10^6 \pm 280.49$	0
	Short Run Emphasis 90°	$1.47 \times 10^6 \pm 321.64$	$1.47 \times 10^6 \pm 287.97$	0
	Short Run Emphasis 135°	$1.47 \times 10^6 \pm 320.93$	$1.47 \times 10^6 \pm 280.49$	0
	Long Run Emphasis 0°	$1.18 \times 10^8 \pm 3.77 \times 10^7$	$6.56 \times 10^7 \pm 2.00 \times 10^7$	0
	Long Run Emphasis 45°	$1.18 \times 10^8 \pm 3.77 \times 10^7$	$6.56 \times 10^7 \pm 2.00 \times 10^7$	0
	Long Run Emphasis 90°	$1.18 \times 10^8 \pm 3.77 \times 10^7$	$6.56 \times 10^7 \pm 2.00 \times 10^7$	0
	Long Run Emphasis 135°	$1.18 \times 10^8 \pm 3.77 \times 10^7$	$6.56 \times 10^7 \pm 2.00 \times 10^7$	0
**GLDM**	Zone Size Variance	$3.28 \times 10^7 \pm 2.71 \times 10^4$	$3.28 \times 10^7 \pm 5.33 \times 10^3$	0.02

Table S6. Texture features with significant statistical differences between malignant and normal cases in the GRRI ROI, including mean, standard deviation, and t-test results.

Classification	Features	Mean Normal ± SD	Mean Malignant ± SD	p-Value
**Intensity**	Mean Intensity	0.65 ± 0.11	0.56 ± 0.08	0
	Median Intensity	0.69 ± 0.16	0.54 ± 0.11	0
	Standard deviation Intensity	0.17 ± 0.06	0.12 ± 0.06	0
	Percentile 90	0.81 ± 0.11	0.70 ± 0.13	0
	Skewness Value	-0.43 ± 1.24	0.76 ± 1.16	0
	Kurtosis Value	3.69 ± 3.05	5.16 ± 3.69	0.01
**GLCM**	Contrast 0°	4.53 ± 1.71	2.77 ± 1.28	0
	Contrast 45°	6.49 ± 2.44	4.00 ± 1.84	0
	Contrast 90°	5.10 ± 2.15	3.00 ± 1.60	0
	Contrast 135°	6.53 ± 2.56	3.95 ± 1.88	0
	Homogeneity 0°	1.00 ± 0.00	1.00 ± 0.00	0
	Homogeneity 45°	1.00 ± 0.00	1.00 ± 0.00	0
	Homogeneity 90°	1.00 ± 0.00	1.00 ± 0.00	0

	Homogeneity 135°	1.00 ± 0.00	1.00 ± 0.00	0
	Dissimilarity	53664.78 ± 15545.94	43023.49 ± 10973.09	0
	Entropy	0.11 ± 0.04	0.13 ± 0.03	0
	Sum Of Squares	$6.65 \times 10^6 \pm 2.51 \times 10^6$	$4.06 \times 10^6 \pm 1.89 \times 10^6$	0
	Variance	$7.36 \times 10^8 \pm 2.14 \times 10^7$	$7.16 \times 10^8 \pm 1.56 \times 10^7$	0
	Inverse Difference Normalized	$1.47 \times 10^6 \pm 372.70$	$1.47 \times 10^6 \pm 252.47$	0
	Sum Average	$3.85 \times 10^6 \pm 1.46 \times 10^5$	$3.72 \times 10^6 \pm 1.10 \times 10^5$	0
	Sum Entropy	$-3.00 \times 10^7 \pm 4307.65$	$-3.00 \times 10^7 \pm 2796.77$	0
	Sum Variance	$2.18 \times 10^{19} \pm 1.64 \times 10^{18}$	$2.03 \times 10^{19} \pm 1.21 \times 10^{18}$	0
	Difference Entropy	$-3.00 \times 10^7 \pm 4348.84$	$-3.00 \times 10^7 \pm 2716.71$	0
	Difference Variance	$3.31 \times 10^6 \pm 1.29 \times 10^6$	$2.02 \times 10^6 \pm 1.02 \times 10^6$	0
**GLRLM**	High Gray Level Run Emphasis 0°	0.00 ± 0.00	0.00 ± 0.00	0
	High Gray Level Run Emphasis 45°	0.00 ± 0.00	0.00 ± 0.00	0
	High Gray Level Run Emphasis 90°	0.00 ± 0.00	0.00 ± 0.00	0
	High Gray Level Run Emphasis 135°	0.00 ± 0.00	0.00 ± 0.00	0.01
**GLDM**	Zone Percentage 0°	0.00 ± 0.00	0.00 ± 0.00	0
	Zone Percentage 45°	0.00 ± 0.00	0.00 ± 0.00	0
	Zone Percentage 90°	0.00 ± 0.00	0.00 ± 0.00	0
	Zone Percentage 135°	0.00 ± 0.00	0.00 ± 0.00	0
	Zone Entropy	$-8.32 \times 10^7 \pm 11950.04$	$-8.32 \times 10^7 \pm 7374.40$	0
	High Gray Level Zone Emphasis 0°	0.00 ± 0.00	0.00 ± 0.00	0
	High Gray Level Zone Emphasis 45°	0.00 ± 0.00	0.00 ± 0.00	0
	High Gray Level Zone Emphasis 90°	0.00 ± 0.00	0.00 ± 0.00	0
	High Gray Level Zone Emphasis 135°	0.00 ± 0.00	0.00 ± 0.00	0
